# Novel Pathogenic Variants of the AIRE Gene in Two Autoimmune Polyendocrine Syndrome Type I Cases with Atypical Presentation: Role of the NGS in Diagnostic Pathway and Review of the Literature

**DOI:** 10.3390/biomedicines8120631

**Published:** 2020-12-19

**Authors:** Luigia Cinque, Cristina Angeletti, Alfredo Orrico, Stefano Castellana, Lucia Ferrito, Cristina Ciuoli, Tommaso Mazza, Marco Castori, Vito Guarnieri

**Affiliations:** 1 Division of Medical Genetics, Fondazione IRCCS Casa Sollievo della Sofferenza, 71013 San Giovanni Rotondo, Italy; l.cinque@operapadrepio.it (L.C.); m.castori@operapadrepio.it (M.C.); 2 UOC Pediatrics and Neonatology, POU AV2, 60122 Senigallia, Italy; Cristina.Angeletti@sanita.marche.it (C.A.); lucia.ferrito@gmail.com (L.F.); 3 Molecular Diagnosis and Characterization of Pathogenic Mechanisms of Rare Genetic Diseases, Azienda Ospedaliera Universitaria Senese, 53100 Siena, Italy; a.orrico@ao-siena.toscana.it; 4 Clinical Genetics, ASL Toscana SudEst. Ospedale della Misericordia, 58100 Grosseto, Italy; 5 Unit of Bioinformatics, Fondazione IRCCS Casa Sollievo della Sofferenza, 71013 San Giovanni Rotondo, Italy; s.castellana@operapadrepio.it (S.C.); t.mazza@operapadrepio.it (T.M.); 6 Department of Medical, Surgical and Neurological Sciences, UOC Endocrinology, Azienda Ospedaliera Universitaria Senese, 53100 Siena, Italy; c.ciuoli@ao-siena.toscana.it

**Keywords:** APS-1, mucocutaneous candidiasis, chronic hypoparathyroidism, Addison disease, next generation sequencing

## Abstract

*Background*. Autoimmune polyglandular syndrome type 1 (APS-1) with or without reversible metaphyseal dysplasia is a rare genetic disorder due to inactivating variants of the autoimmune regulator, *AIRE,* gene. Clinical variability of APS-1 relates to pleiotropy, and the general dysfunction of self-tolerance to organ-specific antigens and autoimmune reactions towards peripheral tissues caused by the underlying molecular defect. Thus, early recognition of the syndrome is often delayed, mostly in cases with atypical presentation, and the molecular confirm through the genetic analysis of the *AIRE* gene might be of great benefit. *Methods*. Our methods were to investigate, with a multigene panel next generation sequencing approach, two clinical cases, both presenting with idiopathic hypoparathyroidism, also comprising the *AIRE* gene; as well as to comment our findings as part of a more extensive review of literature data. *Results*. In the first clinical case, two compound heterozygote pathogenic variants of the *AIRE* gene were identified, thus indicating an autosomal recessive inheritance of the disease. In the second case, only one *AIRE* gene variant was found and an atypical dominant negative form of APS-1 suggested, later confirmed by further medical ascertainments. *Conclusions*. APS-1 might present with variable and sometimes monosymptomatic presentations and, if not recognized, might associate with severe complications. In this context, next generation diagnostics focused on a set of genes causative of partially overlapping disorders may allow early diagnosis.

## 1. Introduction

**Figure 1:**
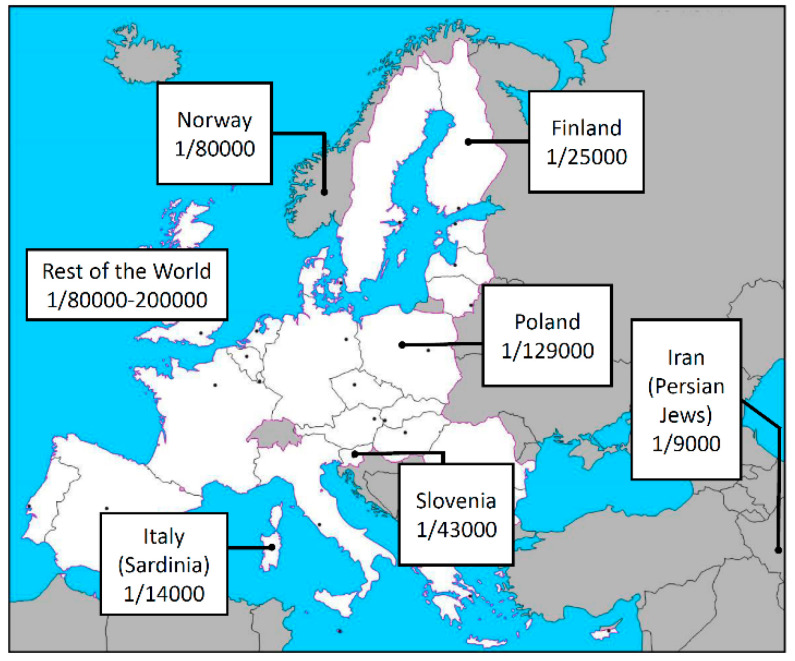
Prevalence of the APS-1 in different European countries and in the rest of the world.

Autoimmune polyglandular syndrome type 1 (APS-1) with or without reversible metaphyseal dysplasia, also known as Autoimmune PolyEndocrinopathy, Candidiasis, and Ectodermal Dystrophy or dysplasia (APECED, MIM 240300), is a rare disorder affecting 1/2–3 million newborns with a variable prevalence ranging from 1/9000 to 1/200,000 in different countries (Figure 1) [1,2,3,4]. The syndrome is a monogenic autoimmune disease mainly characterized by chronic mucocutaneous candidiasis (MC), chronic hypoparathyroidism (CH), and Addison disease (AD) which represents the main key features of the clinical spectrum [5]. Instead, the first manifestation is usually the MC with an onset often before the 5 ys, followed by CH, while AD is usually the latest symptom to appear between 5 and 15 years [5,6,7,8]. Usually, the diagnosis is established by (i) the presence of two or all three these features, or (ii) the presence of only one key feature along with a sibling previously diagnosed with APS1-1, and/or (iii) the presence of interferon-alpha or omega auto-antibodies [9]. However, clinical variability is wide and includes a plethora of additional pleiotropic and secondary manifestations [6], so that cases with ambiguous presentation are not infrequent and might benefit of a molecular confirm by the genetic sequencing of the causative gene.

APS-1 is due to inactivating deleterious variants in the autoimmune regulator, *AIRE,* gene (NM_000383) [10], located on 21q22, causing a general dysfunction of self-tolerance to organ-specific antigens [11,12]. Autosomal recessive of the disease as a unique model of inheritance of APS-1 is overcome. Evidences suggest that APS-1 is inherited either in autosomal recessive or dominant negative fashion [13]. Classic form is inherited as an autosomal recessive trait, with homozygous or compound heterozygous *AIRE* deleterious variants, often clustered at the N-terminus of the protein, which contains a CARD (CAspase Recruitment Domain) motif [14]. Non-classical APS-1 form is due to single heterozygous variants, mainly lying within the PHD1 (Plant Homeodomain) zinc finger motif, acting with a dominant negative effect, i.e., impairing the efficiency of the wild type allele. In autosomal dominant APS-1, the associated phenotype tends to be milder, and with marked intrafamilial and interfamilial variability [14,15].

Here we present two clinical cases both manifesting a different APS-1 spectrum: the proband of the first family showed CH and his sister CH and cutaneous exantema. In the second family, the proband showed only the CH. NGS was done and pathogenic variants of the *AIRE* gene identified, contributing to a definitive diagnosis of APS-1 in both cases. Finally, we discuss our data in the context of an overall revision of literature data.

## 2. Materials and Methods

Molecular studies carried out in this work were based on routine clinical care, thus not requiring any IRB approval, but, as established by the Italian Privacy Laws, only the approved informed consent signed by the patients.

### 2.1. Patients.

*Clinical subject 1.* The proband (II:2, Figure 1A) was a 12-year-old boy, born at term by Cesarean section from an uneventful pregnancy, with a birth weight of 3500 g. Birth parameters and psychomotor development were normal. Family history was positive for febrile seizures. At 8 years he was admitted in the emergency room for fever seizures associated to loss of consciousness, generalized hypertonia, and trismus. At the arrival, the boy was in sopor condition, although he reacted to stimuli. Vital signs were present but coexisted with an inflammation of the upper respiratory tract. Blood chemistry was the following: low serum Ca (4.5 mg/dL, nr: 8.1–10.4), high phosphorus (P: 10.9 mg/dL, nr 4.0–7.0), and low parathyroid hormone (PTH: 1 pg/mL, nr: 14–79). Intravenous therapy with calcium gluconate and then oral therapy with calcium carbonate and calcitriol led to a gradual improvement. Kidney and liver parameters were normal, as well as VitD3, adenocorticotropic hormone (ACTH), cortisol, sodium and potassium, and thyroid autoimmunity. Karyotype and FISH for velocardiofacial (22q11.2 microdeletion) syndrome gave normal results. At the follow-up, two weeks later, plasma Ca levels were stable and the calcium carbonate and calcitriol reduced. Six months later, premature pubarche was observed (puberal stage: P2, G1, testis volume 2 mL, bilateral, estimated by orchimetry, although TSH, ACTH, LHRH, 17-OH progesterone at the basal and after ACTH test were normal and autoimmunity for thyroid and coeliac disease were negative. The genetic analysis was requested when the proband was 10 years and a similar CH episode occurred in the sister.

The proband’s younger sister (II:1, Figure 2A) was born at term by Cesarean section from an uneventful pregnancy, with a birth weight of 3000 g, and a normal perinatal period and psychomotor development. At 11 months, she was admitted for urinary infection (*Escherichia coli* 3 × 106 UFC), associated to a diffuse maculo-papular exanthema, mostly on the face and trunk. After an early worsening, the situation resolved with an anti-histaminic therapy; at that time the serum Ca level was normal (9.1 mg/dL, nr: 8.1–10.4). At 3 years and 7 months she was admitted for a first critic episode of trunk and limbs hypertonia, ocular retroversion with no response to external stimuli that, however, resolved spontaneously. Shortly after, a second episode of limbs hypertonia, left deviation of the eyeballs, and transitory apnea occurred, and endorectal diazepam was required. Hypocalcemia was found (serum Ca: 4.3 mg/dL, nr: 8.1–10.4), while other blood biochemistry values included hyperphosphoremia (P: 12.2 mg/dL, nr: 4.0–7.0), indosable PTH, while kidney, liver, and thyroid parameters as well as sodium and potassium levels were normal and coeliac disease auto-antibodies negative. Karyotype was normal and the chr 22 FISH analysis result was negative. Therapy with intravenous calcium gluconate was started. After remission of clinical manifestations and calcium levels normalization, oral therapy with calcium carbonate and calcitriol was continued. 

*Clinical subject 2.* The proband (II:1, Figure 2C) was a 20-year-old woman. In her past medical history, she was admitted for the first time at 6 years at a local Hospital in Central Italy where she lived for muscular spams due to a tetanic crisis and severe hypocalcemia (Ca: 4.2 mg/dL, nr: 8.1–10.4; PTH: 3.7 pg/mL, nr: 14–79; P: 10 mg/dL, nr: 4.0–7.0). An initial diagnosis of idiopathic early onset hypoparathyroidism was made. At that time, a polyendocrine evaluation was requested but data are not available. Therapy with calcium gluconate in the 1st day and, subsequently, with calcium carbonate and calcitriol, led to the stabilization of calcium levels (8 mg/dL, nr: 8.1–10.4), although the PTH remained low (5.6 pg/mL, nr: 14–79), while other parameters (creatinine, calciuria, 25-OH Vit D, cortisol as well as thyroid and renal ETG, and X-ray at the skull) were normal. At 9 years, a clinical follow-up showed nephrolcalcinosis that required a reduction in the calcium carbonate and the addition of the hydrochlorothiazide. At a more recent follow-up, at 17 ys, the therapy consisted also of Vit D supplementation, and she showed mild hypoparathyroidism, with the PTH below the normal range, Ca slowly low (7.8 mg/dL, nr: 8.1–10.4), and elevated urinary calcium (416 mg/24 h, nr: 100–250 mg/24 h), while phosphorus, magnesium, and creatinine clearance were in the normal range. Eye examination and MRI brain to detect for basal ganglia calcifications were performed. The presence of GAD, anti IA2, pituitary, adrenal gland, ovary, parietal gastric cells autoantibodies, and celiac disease were excluded. After the identification of the AIRE variant, a possible diagnosis of APS-1 was suggested and an accurate dermatological evaluation requested. Then the patient was found to suffer for vaginal candidiasis and psoriasis lesions at the scalp and at the upper limbs. The father, 42 years, (I:1, Figure 2B) carrying for the same pathogenic AIRE variant, was likewise investigated for the presence of hypoparathyroidism and adrenal insufficiency, or other APS-1 stigmata, but at the moment, he is in a healthy, asymptomatic state, with biochemical parameters in the normal range.

### 2.2. Sample Preparation and Next Generation Sequencing Analysis

Informed consent was obtained from the patients, or parents, in case the proband was underage. DNA extracted from peripheral blood leucocytes (Bio Robot EZ1, Qiagen, Hilden, Germany) was quantified (Qubit spectrophotometer, Thermo Fisher Scientific, Waltham, MA, USA) and sequenced with a HaloPlex gene targeted panel (Agilent Technologies, Santa Clara, CA, USA) designed to selectively capture known genes associated with different forms of calcium/parathormone metabolic disorders (AIRE, AP2S1, CASR, CDC73, CDKN1B, CYP24A1, GATA3, GCM2, GNA11, MEN1, PTH, RET, TBCE, TBX1). Targeted fragments were loaded and sequenced on a MiSeq sequencer (Illumina, San Diego, CA, USA) with a MiSeq Reagent kit V3 300 cycles flow cell. 

### 2.3. Bioinformatic Pipeline

Short-reads were aligned to the GRCh37/hg19 human reference genome using BWA (v.0.7.17). The resulting BAM files were sorted with SAMtools v.1.7 and purged from duplicates using the MarkDuplicates tool of the Picard suite v.2.9. The mapped reads were then realigned with GATK v.3.8 and dropped if exhibiting mapping quality scores of <20 or containing at most 2 nucleotides with a phred score of <30. The GATK’s HaplotypeCaller tool was used to identify variants. These were subsequently annotated using ANNOVAR. 1000 Genomes, dbSNP v.151, GO-ESP 6500, ExAC, TOPMED, GnomAD, NCI60, COSMIC were queried to determine the allelic frequency of variants. ClinVar, HGMD, LOVD, or SIFT, PolyPhen2, LRT, MutationTaster, MutationAssessor, FATHMM, PROVEAN, VEST3, MetaSVM, MetaLR, M-CAP, CADD, DANN, fathmm-MKL, Eigen, and GenoCanyon were used to report or infer, computationally, the pathogenicity of variants. fitCons, GERP++, phyloP100way, phyloP20way, phastCons100way vertebrate, phastCons20way mammalian, and SiPhy 29way were used to report their evolutionary conservation scores. Prioritization of the variants was done following the American College of Medical Genetics and Genomics/Association for Molecular Pathology (ACMGG/AMP) criteria [16]. Variants sorted as “benign” and “likely benign” were excluded. Remaining variants were classified as pathogenic, likely pathogenic, or of uncertain significance (VUS) by the following criteria: (i) null variant (nonsense, frameshift, deletion, insertion, canonical ±1 or ±2 splicing site) in genes previously described as disease-causing by haploinsufficiency or loss-of-function; (ii) variant considered as a mutational hot spot and/or lying in well-established functional domain; (iii) variant absent in allele frequency population databases; (iv) variant reported in allele frequency population databases, but with a minor allele frequency (MAF) significantly lower than expected for the disease; (v) variant annotated as pathogenic in ClinVar and/or LOVD; (vi) variant co-segregation with disease in multiple affected family members; (vii) well-established in vitro or in vivo functional studies supportive of a damaging effect on the gene or gene product. Common (MAF > 0.01) and synonymous variants were discarded.

### 2.4. Multialignment of AIRE Orthologous Sequences

Selected AIRE orthologous aminoacidic sequences were downloaded from the Ensembl database (http://www.ensembl.org/Homo_sapiens/Info/Index) and multialigned using the ClustalW online tool (https://www.genome.jp/tools-bin/clustalw) using default parameters.

## 3. Results

### 3.1. NGS

**Figure 2:**
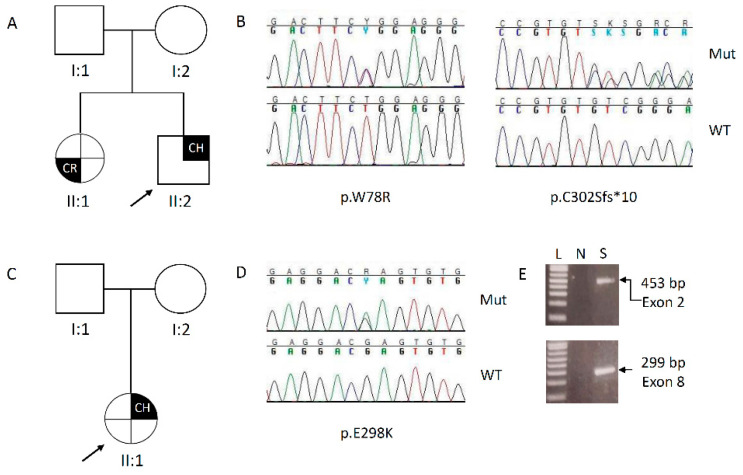
(**A**–**C**): Pedigree of the families under study. The arrow indicates the proband; (**B**–**D**): elechropherograms showing the pathogenic variants identified. For each case, on the top the mutated sequence, on the bottom the wild type; CH: chronic hypoparathyroidism; CR: cutaneous rush; L = ladder; N = negative PCR reaction; S = sample.

*Subject 1.* Targeted sequencing on proband’s DNA identified two different variants of the *AIRE* gene, both in heterozygosity (Figure 2B). The first variant, located in the exon 2, changes a Trp to Arg at the position 78 [c.232T>C, p.(Trp78Arg)], it was reported in dbSNP (rs179363880) and in the gnomAD databases with a very low frequency (MAF = 7.7 × 10^−6^) and classified by ClinVar (https://www.ncbi.nlm.nih.gov/clinvar/) as pathogenic. Moreover, in the exon 8, a novel frameshift deletion of a GT dinucleotide in a stretch of three, with a premature stop codon at the position 312 [c.905_906delGT, p.(Cys302Serfs*10)], was detected. Varsome online tool (https://varsome.com/) was interrogated and, following the interpretation criteria, the p.(Trp78Arg) variant was classified as “likely pathogenic”, while the p.(Cys302Serfs*10) was classified as “pathogenic”. Segregation analysis performed on DNA belonging to the available relatives, revealed co-segregation of both the variants in the younger affected sister, while the unaffected parents carried only one mutation: the father carried the aminoacidic change, while the mother carried the intragenic deletion (Figure 1A). This result confirmed the classic autosomal recessive inheritance of the disease.

*Subject 2.* On proband’s DNA, the NGS detected the recurrent nucleotide change in the exon 8, close to the deletion identified in the subject 1 (Figure 2D). The c.892G>A, p.(Glu298Lys) variant was present in dbSNP (rs763636007) and gnomAD, with a likewise low frequency (MAF = 7.9 × 10^−6^), but it was classified as Variant of Uncertain Significance (VUS) by ClinVar. Conversely, Varsome classified as “likely pathogenic” the variant, that, by segregation analysis, was found on the DNA of the apparently unaffected father (Figure 2C).

All the variants were confirmed by direct PCR on proband’s DNA and Sanger sequencing as part of our quality assurance guidelines.

### 3.2. ClustalW Analysis

**Figure 3:**
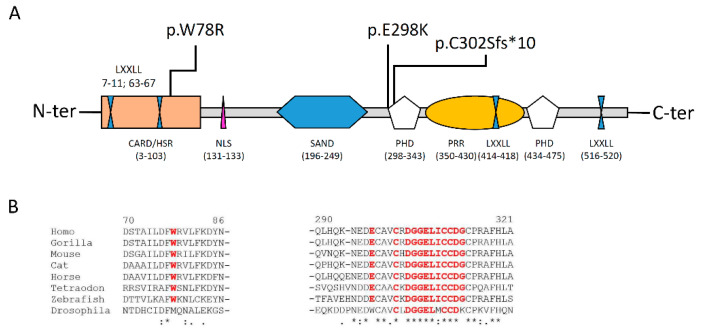
(**A**): Structure of the *AIRE* protein and description of the different domains (please see to text for details); (**B**): Homolog sequence multialignment showing the level of conservation of the aminoadic sequence in different orthologues. In red the residues resulted in mutation or the sequence following the frameshift deletion.

The p.(Trp78Arg) variant lies in the CARD/HSR domain, while the other two, close to one of each other, both fall in the PHD1 domain (Figure 3A). Homolog sequence multialignment showed that the W78 and the E298 are conserved in fishes, while the sequence across and after the C302 residue is conserved even in insects (Figure 3B), thus strongly suggesting the importance of this aminoacidic sequence for the protein function.

## 4. Discussion

APS-1 is an autoimmune syndrome affecting endocrine and not endocrine organs. Different symptoms reflect the variable organs involvement and can be divided in major categories:

- Mucosa infection/inflammation: chronic MC;
- Impaired endocrine organ functions: CM, AD, hypothyroidism, panhypopituitarism;
- Ectodermal dystrophy (nail distrophy, enamel hypoplasia, alopecia, vitiligo, keratopathy).

Definitive diagnosis is based on the presence of at least two of the symptoms of the triad (MC, CH, and AD), or on one clinical sign in a family with at least one sibling already diagnosed with APS-1. The dosage of interferon omega and alpha can help the diagnosis, although it is not conclusive [9]. The presence of two or all the following three clinical signs defines the “classic” APS-1 form:

- Chronic Mucocutaneous Candidiadis: it is present in up to 80% of all the patients aged one to three years and is mostly located at the oral mucosa and esophagus while, less frequently, it affects vaginal or intestinal and nail mucosa. Chronic infection makes the mucosa susceptible to squamous cell carcinoma of the mouth or of the esophagus in 5% of cases [17,18];
- Chronic Hypoparathyroidism is present in up to 80–90% of patients aged 10–15 years and characterized by paresthesias and tetany (mimicking an epileptic seizure) with hypocalcemia, hypophosphoremia, and a low PTH level. Autoantibodies NALP5 against parathyroids glands can be present in 11–38% of patients [19];
- Addison disease is the third late sign to appear, at around 13–14 years of age, and it consists in the nearly complete atrophy of the adrenal gland. It manifests with asthenia, hypotension, weight loss, hyperpigmentation of the skin, and mucosa [20]. The main biochemical feature is the lack of cortisol secretion after the ACTH stimulation test. This is due to the presence of autoantibodies against the adrenal cortex (ACA) and the 21-OH hydroxylasis enzyme. These antibodies are specific of the disease being present in up to 50% of patients with MC and CH [21].

Despite the specific triad of symptoms (MC, CH, and AD) paradigmatic of a definitive APS-1 diagnosis, many other clinical conditions can manifest, depending on the broad gamut of the aberrantly overexpressed antigens induced by the AIRE protein towards different peripheral tissues. Thus, the APS-1 clinical spectrum can be variable and includes up to 30 different symptoms, 90% of them affecting non-endocrine organs such as ([22] and references therein):

- Pancreas: diabetes mellitus I, up to 5% of patients;
- Liver: hepatitis in up to 18% of cases that sometimes it can outcome in cirrhosis;
- Stomach: chronic atrophic gastritis, with/without pernicious anemia, 13–27%. Patients having also intestinal metaplasia are at a high risk to develop gastric cancer;
- Lung: pneumonitis, often misdiagnosed as bronchitis or asthma, due to the presence of cough;
- Thyroid: Hashimoto thyroiditis;
- Gut: malabsorption;
- Kidneys: interstitial tubulonephritis and nephrolithiasis;
- Spleen: asplenia;
- Gonads: hypergonadotrophic hypogonadism, 24–60% of cases, as premature ovarian failure in females under 30 years of age;
- Salivary and lacrimal glands (Sjogren’s like syndrome).

As obvious consequence, the clinical management requires a multidisciplinary approach, since non-classical forms sometimes represent a diagnostic challenge for the physician.

Instead, such high phenotypic heterogeneity can cause a delay of the final diagnosis for several years, with a high risk to develop other, sometimes irreversible, sequelae. Early diagnosis and recognition of clinical signs at their first appearance is extremely useful for the follow-up of patients in order to set the adequate therapy and avoid further complications. As stated above, APS-1 can associate to a high risk of (multiorgan) adrenal, renal, or liver failure, life-threatening hypocalcemic seizures, or POF, this latter affecting up to 60% of APS-1 women before 30 years of age [4] and, in general, to detrimental long-term outcomes. More importantly, compared to the general population, APS-1 patients have a higher risk of developing gastric adenocarcinoma and squamous-cell carcinoma of mouth and esophagus [23]. Finally, the early detection of a genetic pathogenic variant may allow to estimate the recurrence risks in the offspring (1/4 in the classical forms and 1/2 in the non-classical forms), a necessary information for the families, although to predict the phenotype from a given AIRE gene variant may be difficult, because of the great variability of the phenotypical expression, especially in non-classical forms. However, a recent work in an American APS-1 cohort reported a delay of up to seven years for the definitive diagnosis, since only the 20% of patients showed classic dyad as first consecutive signs, while 80% manifested other symptoms [24].

### 4.1. Differential Diagnosis

APECED, and the AIRE gene analysis, should be considered in the differential diagnosis with other autoimmune polyendocrine syndromes, especially with hypoparathyroidism and/or adrenal insufficiency. On the other hand, the involvement of the immune system in the pathogenesis of endocrine pathologies, extensively documented, is more complex. It may involve genetic determinants that play a most important role, as for the case of AIRE gene mutations in APECED or for the MICA5.1 allele in many Addison’s cases [25], but, more often, seems to recognize a multifactorial mechanism due to the contribution of other genetic or environmental events, which explains the highly variable clinical pictures of these conditions [26].

### 4.2. AIRE: Gene and Protein

Autoimmune regulator, AIRE, gene maps on chr 21q22 and encodes a 57.5 kDa protein of 545 residues, from a 2690 bp mRNA. The protein plays the main role of transcription factor of many TSAs involved in tolerance immunity. Although expressed ubiquitously in the early embryogenesis [27], during the development, AIRE expression is restricted to the thymus and, specifically, in the Medullary Thymic Epithelial Cells, as well as in other secondary lymphoid organs [28].

**Figure 4:**
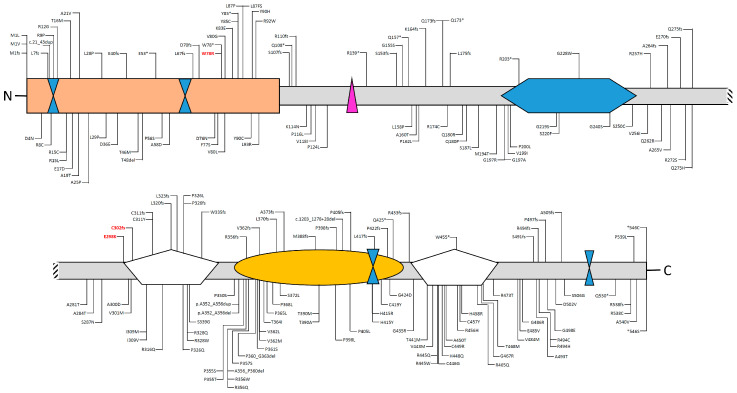
Deleterious (pathogenic and likely pathogenic) variants and variants of unknown significance (VUS) identified in the AIRE gene and their locations within the different domains of the protein. Deleterious variants are shown above, while VUS is below the protein structure. Data have been downloaded from ClinVar (https://www.ncbi.nlm.nih.gov/clinvar/?term=aire[gene]). Please note that large genomic deletions, benign, and synonymous variants are not shown.

The protein is structured in different functional domains [14] (Figure 3 and Figure 4):

- One CARD/homogeneously staining region HSR (aa 3-103), involved in AIRE dimerization and in the chromatin binding;
- Two zinc fingers of plant homeodomain (PHD1 and 2) type (aa 298-341 and 433-476, respectively) involved in the recruitment of chromatin related proteins and in AIRE transcriptional activity;
- One DNA binding domain (SAND) that is engaged in promoting a protein–protein interaction with a transcriptional repressive complex;
- Four nuclear receptor binding LXXLL modules, involved in promoting gene transcription;
- One inverted LXXXLL domain, as transcription coactivator;
- One proline rich region (PRR) involved in promoting gene transcription.

Once translated into the cytoplasm, the protein is translocated into the nucleus where it forms homodimers and tetramers through the CARD domain [14]. Evidences suggest that phosphorylation at several serine and/or threonine residues of the CARD domain, by cAMP-dependent PKA and/or PKC, is critical to trigger the dimerization [14]. Studies on monoallelic variants of the CARD domain, altering the monomer folding and, in turn, impairing the dimerization, were able to establish the alternative dominant negative inheritance of the disease [13]. Into the nucleus, AIRE, plays different functions:

- It contributes to the synthesis of Tissue Specific Antigens, TSA [29,30];
- It binds to different proteins, such positive elongation factors (P-TEFb) and heterogeneous ribonucleoproteins (HNRPL and RNA pol II) [31];
- It is involved in the modulation of chromatin mediated by the interaction with a specific complex (ATF7IP-MBD1) in order to induce the CpG demethylation of TSAs genes [32].

Deleterious variants of the AIRE gene contribute to autoimmunity because of a decrease of central tolerance; instead, loss of the *AIRE* function implies that autoreactive T cells could survive and respond to “self” proteins, leading to a production of autoantibodies against many peripheral tissues and, then, inducing their self-destruction. Up to date, ClinVar reported 101 pathogenic and 114 variants of uncertain significance (VUS) scattered out throughout the entire coding sequence, although the majority are located into the CARD or PHD domains. 

Genetic studies on different APS-1 cohorts were able to possibly define a founder effect of specific pathogenic variants in different countries, such as the p.(Tyr85Cys) in Persian Jews or the p.(Arg257*) in Finns [1,2,3,4]. In the same way, Italian cohorts show a geographical distribution of some recurrent variants with a potential founder effect, in Sardinians, where the recurrent mutation is the p.(Arg139*), Apulia, with the p.(Trp78Arg) (identified in the subject I) and the p.(Gln358*), and the p.(Arg257*) in Veneto [2,33,34]. However, the overall distribution of pathogenic and VUS showed a clusterization with more than 10% of the occurrences at the exons 1, 2, and 10. However, due the above-mentioned high phenotypic heterogeneity, no genotype–phenotype correlation was established. It can be added that even siblings carrying the same mutation might have a great clinical variability [35]. This would also suggest the presence of genetic modifiers that might amplify or reduce the susceptibility of peripheral organs to TSAs, as recently supposed [36].

### 4.3. Pathogenic Effect of the p.(Trp78Arg) and p.(Gly2898Lys) Variants

With regard to the pathogenic variants reported in this work, literature data described several times the p.(Trp78Arg) in classic APS-1 forms, always in homozigosity or as compound heterozigosity [33,34,37,38,39]. It was also functionally tested by a two-hybrid system assay that showed how the change at Trp78 residue altered the folding of the protein and impaired the proper homodimerization of the complex [39]. As the p.(Trp78Arg), several other deleterious variants were identified in the CARD/HSR domain and tested for their impairing effect on the functional complex and the vast majority was classified as recessive, a few as dominant negative [13].

PHD-type zinc fingers play a crucial role for the *AIRE* function. It was demonstrated that PHD fingers are implicated in nuclear dots formation, in transactivational capacity, histone recognition, and epigenetic mechanism [40]. Chignola et al. showed that a PHD1 finger is important for the direct interaction between *AIRE* and TSAs through binding to the histone H3 tails non-methylated at K4 (H3K4me0) [40]. In particular, they determined the three-dimensional solution structure of the complex composed by *AIRE*-PHD1, a construct consisting of wild-type residues Gln293-Glu354, with the first 10 aminoacids of histone H3 (H3K4me0). By a site-direct mutagenesis, they verified the specificity of the interaction and proved the importance of Glu298 residue in the formation of polar contacts between K9 and the side chains of Glu298. Indeed, fluorescence binding experiments and ITC performed on p.(Glu298Ala) mutants indicated a more than five-fold reduction in the binding affinity, supporting the notion that transient electrostatic interactions occur in the complex [40]. As a further support, Oftedal and colleagues later confirmed that isoform carrying a 298Lys residue exerted a dominant negative effect on the wild-type counterpart as many other variants falling into the PHD1, that, unlike from the ones of the CARD domain, were mostly dominant negative, rather than recessive [13]. 

Based on the large series of experiments focused on the potential impact of the variants, on the overall function of the *AIRE* protein, and on the localization in the CARD or PHD domains, it was possible to classify the mutations in two different groups:

i. Recessive, mostly located in the CARD/HSR domain with a direct impact on the homo-tetramerization of the complex; mutations falling in this group cause a “classic phenotype”;
ii. Dominant-negative, mostly located in the PHD1 domain and affecting the transactivation-transcription function; mutations falling in this group cause a, often, milder “non-classical phenotype” [9].

### 4.4. Genetic Analysis as Helpful Tool for a Definitive Diagnosis

With regard to the work here reported, the role of the NGS was decisive in both the cases that were featured by a not clear, unequivocal clinical landscape. Instead, in family 1, the proband and his sister showed only one sign of the classic triad (the CH); actually, the sister also manifested the cutaneous rush, that, however, was observed in selected American cohorts and considered part of the disorder by some authors only [24]. Thus, although a suspect of APS-1 was raised, the genetic confirm with the identification of the compound heterozigosity was mandatory for a definitive diagnosis. Similarly, the proband of family 2 did present with early onset CH without other signs. Instead, only after the genetic test, and a further dermatological investigation that revealed MC and psoriasis, 14 years after the first manifestation, a conclusive diagnosis of APS-1 was established. We can add, in this latter case, that, in accord with the late onset of other symptoms, we cannot rule out that other physiological compensatory mechanisms or genetic modifiers (as previously supposed) could contribute to the late penetrance associated to this variant [13] and to the apparently healthy state of the father. This would be also in line with the great variability of phenotypic manifestation accompanying this disease.

Our results stress the use of NGS in helping the clinical APS-1 diagnostic approach, mostly in patients manifesting a not classic phenotype that, as reported above, might represent up to 80% of the cases [24]. In this view, the molecular confirmation by multigene panels intersecting individual’s clinical presentation becomes of paramount importance in accelerating the diagnosis and, in turn, the early correct patient management in order to avoid the worsening of the symptoms.

## 5. Conclusions

We report on a novel compound heterozygote and on a dominant negative mutation of the AIRE gene in patients affected by atypical APS-1 forms. Our work enlarges the mutational spectrum of the AIRE gene and confirms inheritance heterogeneity at the same locus for APS-1, as well as demonstrating the utility of NGS in the early diagnosis of this disorder.

## References

[1] Zlotogora J., Shapiro M.S. (1992). Polyglandular autoimmune syndrome type I among Iranian Jews. J. Med. Genet..

[2] Rosatelli M.C., Meloni A., Meloni A., Devoto M., Cao A., Scott H.S., Peterson P., Heino M., Krohn K.J., Nagamine K. (1998). A common mutation in Sardinian autoimmune polyendocrinopathy-candidiasis-ectodermal dystrophy patients. Hum. Genet..

[3] Wolff A.S., Erichsen M.M., Meager A., Magitta N.F., Myhre A.G., Bollerslev J., Fougner K.J., Lima K., Knappskog P.M., Husebye E.S. (2007). Autoimmune polyendocrine syndrome type 1 in Norway: phenotypic variation, autoantibodies, and novel mutations in the autoimmune regulator gene. J. Clin. Endocrinol. Metab..

[4] Bruserud Ø., Oftedal B.E., Landegren N., Erichsen M.M., Bratland E., Lima K., Jørgensen A.P., Myhre A.G., Svartberg J., Fougner K.J. (2016). A Longitudinal Follow-up of Autoimmune Polyendocrine Syndrome Type 1. J. Clin. Endocrinol. Metab..

[5] Ahonen P., Myllärniemi S., Sipilä I., Perheentupa J. (1990). Clinical variation of autoimmune polyendocrinopathy-candidiasis-ectodermal dystrophy (APS-1) in a series of 68 patients. N. Engl. J. Med..

[6] Perheentupa J. (2006). Autoimmune polyendocrinopathy-candidiasis-ectodermal dystrophy. J. Clin. Endocrinol. Metab..

[7] Betterle C., Greggio N.A., Volpato M. (1998). Clinical review 93: Autoimmune polyglandular syndrome type 1. J. Clin. Endocrinol. Metab..

[8] Meager A., Visvalingam K., Peterson P., Möll K., Murumägi A., Krohn K., Eskelin P., Perheentupa J., Husebye E., Kadota Y. (2006). Anti-interferon autoantibodies in autoimmune polyendocrinopathy syndrome type 1. PLoS Med..

[9] Husebye E.S., Perheentupa J., Rautemaa R., Kämpe O. (2009). Clinical manifestations and management of patients with autoimmune polyendocrine syndrome type I. J. Intern. Med..

[10] Nagamine K., Peterson P., Scott H.S., Kudoh J., Minoshima S., Heino M., Krohn K.J.E., Lalioti M.D., Mullis P.E., Antonarakis S.E. (1997). Positional cloning of the APS-1 gene. Nat. Genet..

[11] Chan A.Y., Anderson M.S. (2015). Central tolerance to self revealed by the autoimmune regulator. Ann. N. Y. Acad. Sci..

[12] Peterson P., Org T., Rebane A. (2008). Transcriptional regulation by AIRE: molecular mechanisms of central tolerance. Nat. Rev. Immunol..

[13] Oftedal B.E., Hellesen A., Erichsen M.M., Bratland E., Vardi A., Perheentupa J., Kemp E.H., Fiskerstrand T., Viken M.K., Weetman A.P. (2015). Dominant Mutations in the Autoimmune Regulator AIRE Are Associated with Common Organ-Specific Autoimmune Diseases. Immunity.

[14] Kumar P.G., Laloraya M., Wang C.Y., Ruan Q.G., Davoodi-Semiromi A., Kao K.J., She J.X. (2001). The autoimmune regulator (AIRE) is a DNA-binding protein. J. Biol. Chem..

[15] De Martino L., Capalbo D., Improda N., Lorello P., Ungaro C., Di Mase R., Cirillo E., Pignata C., Salerno M. (2016). Novel Findings into AIRE Genetics and Functioning: Clinical Implications. Front. Pediatr..

[16] Richards S., Aziz N., Bale S., Bick D., Das S., Gastier-Foster J., Grody W.W., Hegde M., Lyon E., Spector E. (2015). Standards and guidelines for the interpretation of sequence variants: A joint consensus recommendation of the American College of Medical Genetics and Genomics and the Association for Molecular Pathology. Genet. Med..

[17] Rautemaa R., Hietanen J., Niissalo S., Pirinen S., Perheentupa J. (2007). Oral and oesophageal squamous cell carcinoma--a complication or component of autoimmune polyendocrinopathy-candidiasis-ectodermal dystrophy (APECED, APS-I). Oral. Oncol..

[18] Bensing S., Brandt L., Tabaroj F., Sjöberg O., Nilsson B., Ekbom A., Blomqvist P., Kämpe O. (2008). Increased death risk and altered cancer incidence pattern in patients with isolated or combined autoimmune primary adrenocortical insufficiency. Clin. Endocrinol..

[19] Alimohammadi M., Björklund P., Hallgren A., Pöntynen N., Szinnai G., Shikama N., Keller M.P., Ekwall O., Kinkel S.A., Husebye E.S. (2008). Autoimmune polyendocrine syndrome type 1 and NALP5, a parathyroid autoantigen. N. Engl. J. Med..

[20] Burk C.J., Ciocca G., Heath C.R., Duarte A., Dohil M., Connelly E.A. (2008). Addison’s disease, diffuse skin, and mucosal hyperpigmenation with subtle “flu-like” symptoms—A report of two cases. Pediatr. Dermatol..

[21] Winqvist O., Karlsson F.A., Kämpe O. (1992). 21-Hydroxylase, a major autoantigen in idiopathic Addison’s disease. Lancet.

[22] Constantine G.M., Lionakis M.S. (2019). Lessons from primary immunodeficiencies: Autoimmune regulator and autoimmune polyendocrinopathy-candidiasis-ectodermal dystrophy. Immunol. Rev..

[23] Leibowitz G., Amir G., Losses I.S., Eliakim R. (1993). Autoimmune polyglandular failure associated with malabsorption and gastric carcinoid tumour. J. Intern. Med..

[24] Ferre E.M.N., Rose S.R., Rosenzweig S.D., Burbelo P.D., Romito K.R., Niemela J.E., Rosen L.B., Break T.J., Gu W., Hunsberger S. (2016). Redefined clinical features and diagnostic criteria in autoimmune polyendocrinopathy-candidiasis-ectodermal dystrophy. JCI Insightig..

[25] Triolo T.M., Baschal E.E., Armstrong T.K., Toews C.S., Fain P.R., Rewers M.J., Yu L., Miao D., Eisenbarth G.S., Gottlieb P.A. (2009). Homozygosity of the polymorphism MICA5.1 identifies extreme risk of progression to overt adrenal insufficiency among 21-hydroxylase antibody-positive patients with type 1 diabetes. J. Clin. Endocrinol. Metab..

[26] Husebye E.S., Anderson M.S., Kämpe O. (2018). Autoimmune Polyendocrine Syndromes. N. Engl. J. Med..

[27] Gardner J.M., Metzger T.C., McMahon E.J., Au-Yeung B.B., Krawisz A.K., Lu W., Price J.D., Johannes K.P., Satpathy A.T., Murphy K.M. (2013). Extrathymic Aire-expressing cells are a distinct bone marrow-derived population that induce functional inactivation of CD4^+^ T cells. Immunity.

[28] Nishikawa Y., Hirota F., Yano M., Kitajima H., Miyazaki J., Kawamoto H., Mouri Y., Matsumoto M. (2010). Biphasic Aire expression in early embryos and in medullary thymic epithelial cells before end-stage terminal differentiation. J. Exp. Med..

[29] Anderson M.S., Venanzi E.S., Klein L., Chen Z., Berzins S.P., Turley S.J., von Boehmer H., Bronson R., Dierich A., Benoist C. (2002). Projection of an immunological self shadow within the thymus by the aire protein. Science.

[30] Gotter J., Brors B., Hergenhahn M., Kyewski B. (2004). Medullary epithelial cells of the human thymus express a highly diverse selection of tissue-specific genes colocalized in chromosomal clusters. J. Exp. Med..

[31] Anderson M.S., Su M.A. (2016). AIRE expands: New roles in immune tolerance and beyond. Nat. Rev. Immunol..

[32] Waterfield M., Khan I.S., Cortez J.T., Fan U., Metzger T., Greer A., Fasano K., Martinez-Llordella M., Pollack J.L., Erle D.J. (2014). The transcriptional regulator Aire coopts the repressive ATF7ip-MBD1 complex for the induction of immunotolerance. Nat. Immunol..

[33] Meloni A., Perniola R., Faà V., Corvaglia E., Cao A., Rosatelli M.C. (2002). Delineation of the molecular defects in the AIRE gene in autoimmune polyendocrinopathy-candidiasis-ectodermal dystrophy patients from Southern Italy. J. Clin. Endocrinol. Metab..

[34] Cervato S., Mariniello B., Lazzarotto F., Morlin L., Zanchetta R., Radetti G., De Luca F., Valenzise M., Giordano R., Rizzo D. (2009). Evaluation of the autoimmune regulator (AIRE) gene mutations in a cohort of Italian patients with autoimmune-polyendocrinopathy-candidiasis-ectodermal-dystrophy (APS-1) and in their relatives. Clin. Endocrinol..

[35] Bruserud Ø., Oftedal B.E., Wolff A.B., Husebye E.S. (2016). AIRE-mutations and autoimmune disease. Curr. Opin. Immunol..

[36] Proekt I., Miller C.N., Lionakis M.S., Anderson M.S. (2017). Insights into immune tolerance from AIRE deficiency. Curr. Opin. Immunol..

[37] Betterle C., Ghizzoni L., Cassio A., Baronio F., Cervato S., Garelli S., Barbi E., Tonini G. (2012). Autoimmune-polyendocrinopathy-candidiasis-ectodermal-dystrophy in Calabria: clinical, immunological and genetic patterns. J. Endocrinol. Investig..

[38] Capalbo D., Mazza C., Giordano R., Improda N., Arvat E., Cervato S., Morlin L., Pignata C., Betterle C., Salerno M. (2012). Molecular background and genotype-phenotype correlation in autoimmune-polyendocrinopathy-candidiasis-ectodermal-distrophy patients from Campania and in their relatives. J. Endocrinol. Investig..

[39] Meloni A., Fiorillo E., Corda D., Perniola R., Cao A., Rosatelli M.C. (2005). Two novel mutations of the AIRE protein affecting its homodimerization properties. Hum. Mutat..

[40] Chignola F., Gaetani M., Rebane A., Org T., Mollica L., Zucchelli C., Spitaleri A., Mannella V., Peterson P., Musco G. (2009). The solution structure of the first PHD finger of autoimmune regulator in complex with non-modified histone H3 tail reveals the antagonistic role of H3R2 methylation. Nucleic Acids Res..

